# Treatment with high-dose antidepressants severely exacerbates the pathological outcome of experimental *Escherichia coli* infections in poultry

**DOI:** 10.1371/journal.pone.0185914

**Published:** 2017-10-11

**Authors:** Sofie Kromann, Egle Kudirkiene, Lili Li, Ida Thoefner, Elisabeth Daldorph, Jens Peter Christensen, Hecheng Meng, Rikke Heidemann Olsen

**Affiliations:** 1 Department of Veterinary and Animal Sciences, University of Copenhagen, Frederiksberg, Denmark; 2 Research Institute of Food Safety and Nutrition, Jinan University, Guangzhou, People's Republic of China; 3 School of Food Science and Engineering, South China University of Technology, Guangzhou, People’s Republic of China; Ross University School of Veterinary Medicine, SAINT KITTS AND NEVIS

## Abstract

There is an urgent need for novel antibiotics as the current antibiotics are losing their value due to increased resistance among clinically important bacteria. Sertraline, an on-marked anti-depressive drug, has been shown to modify bacterial activity *in vitro*, including increasing the susceptibility of *Escherichia coli* to antibiotics. The aim of the present study was to investigate if the antimicrobial activity of sertraline could be documented under clinical settings, hereunder if sertraline could potentiate the effect of tetracycline in treatment of an experimentally induced ascending infection in poultry. A total of 40 chickens were divided in four groups of 10 chickens each. All chickens were challenged with 4x10^3^ colony forming units (CFU) of a tetracycline resistant *E*. *coli* strain using a surgical infection model, and subsequently treated with either high-dose sertraline, tetracycline, a combination hereof or received no treatment. Seven days post challenge all birds were submitted to necropsy and scored pathologically for lesions. The average lesion scores were significantly higher (P<0.05) in the groups that were treated with high-dose sertraline or high-dose sertraline combined with tetracycline. In conclusion high-dose treatments (four times the maximum therapeutic dose for treating human depression) with sertraline as an adjuvant for treatment of antibiotic resistant *E*. *coli* infections exacerbate the pathological outcome of infection in chickens.

## Introduction

The progressive incline in antimicrobial resistance of clinical important bacteria has led to an increased focus of so-called non-antibiotics, which may be defined as medical compounds whose primary indication for use is non-infectious diseases, but also processes antimicrobial or antimicrobial helper compound effects [1]. Neurotropic medical compounds, such as phenothiazine and selective serotonin reuptake inhibitors (SSRIs) have been largely recognized for their non-antibiotic properties [2–8]. Phenothiazine and derivatives hereof have shown promising results for reversal of antimicrobial properties of multi-resistant Mycobacterium strains [8–13], the causative agent of tuberculosis, which is a serious threat to human health and highly prevalent in developing countries. In industrialized countries the emergence of multi-resistant, clinically important Gram positive and Gram negative bacteria are likely to constitute a health threat to humans and domestic animals [14–16]. Tetracycline is a valued antibiotic in animal husbandry due to its broad-spectrum activities, low toxicity and several oral-based formulations [17,18]. Tetracycline is also used in humane medicine, although the use has decreased in the last decade, likely due to development of better antibiotics and due to a dramatic increase in tetracycline resistant bacteria [17]. The increase in the prevalence of tetracycline resistance in clinically important bacteria in animals is a major problem as it forces the use of other antibiotics for treatment of infectious disease in livestock production. Substituting tetracycline with other antibiotics primarily used for human infections, e.g. cephalosporines would favour selection of cephalosporin-resistant bacteria, which may be directly or indirectly transferred to humans [19,20].

*In vitro*, sertraline, an SSRI compound, has shown synergy with tetracycline; hence increased the sensitivity of tetracycline-resistant *Escherichia coli* strains to tetracycline [2,3,21]. If so, the combined treatment with sertraline and tetracycline may allow for the current use of tetracycline even in infections caused by tetracycline resistant organisms in livestock production, and even in human infections in which the infective organism has faced resistance to the last line of antibiotics, such as colistin [22].

Therefore, the aim of the present study was to assess the effect of a combinational treatment of sertraline and tetracycline in an experimentally induced infection caused by high-tetracycline resistant *E*. *coli* in a chicken model of ascending bacterial infection.

## Material and methods

### Bacterial model organism

The strain of *E*. *coli* APEC_O2 (NCBI association nr. GCA_001620375.1) was used as challenge strain. The strain originates from a joint of a diseased chicken with arthritis [23]. The strain carries two large plasmids, one being a virulence-associated plasmid [24] and the other being an antimicrobial resistance plasmid, encoding resistance towards a number of antibiotics and heavy metals, including tetracycline [25]. The virulence plasmid has been shown to increase virulence in an avian air sac model [26] as well as contributing to enhanced killing of embryos, growth in human urine and colonization of the murine kidney [27]. The present study is the first to evaluate virulence of *E*. *coli* APEC_O2 in an avian ascending model.

### Minimal inhibitory concentration (MIC) determinations and synergy assessment

The minimum inhibitory concentration (MIC) of APEC_O2 for sertraline (Sigma-aldrich, Denmark) and tetracycline (Sigma-aldrich, Denmark), respectively, were determined by serial broth dilutions method [28].

### Effect of sertraline on the MIC of tetracycline

Synergistic effect of sertraline on tetracycline activity against *E*. *coli* APEC O2 was evaluated by checkerboard method with 96-well microtiter plates using MH broth, as described elsewhere [29]. For each combination, the fractional inhibitory concentration (FIC) was calculated as the MIC of the tetracycline in combination with sertraline divided by the MIC of the tetracycline alone and likewise for sertraline. The FIC indexes were derived from summation of individual FICs. The efficacy of the combination effect was interpreted from the FIC index as follows: synergism was defined as an FIC index ≤ 0.5; indifference as 0.5 < FIC index ≤ 4; and antagonism as FIC index > 4 [28].

### Experimental groups and housing

Forty brown layers (Bowan brown) were purchased from a commercial layer farm. At 42 weeks of age the layers were transferred from the farmer to the experimental housing unit at University of Copenhagen. They were allowed three weeks of acclimatisation before the experimental infections. During the acclimatisation period the birds were monitored for wellbeing, normal avian behaviour and appetite. Birds were kept free range on deep litter in separate floor pens (2.4 × 2.4 m) with unlimited access to nests, perches and dust baths. Water and commercially layer feed was provided *ad libitum* in all groups throughout the experiment. All procedures performed on the birds were approved and licensed by the Danish Animal Experiments Inspectorate (license no. 2013-15-2934-00923).

### Preparation of challenge inoculum

*E*. *coli* APEC_O2 was stored at—80°C in Brain and Heart Infusion (BHI) broth (Oxoid, Basingstoke, UK) in 15% (v/v) glycerol until needed. The day before inoculation the strain was grown overnight in BHI broth. The overnight culture of the strain was diluted to 4×10^4^ CFU, and 0.1 ml of the diluted inoculum were aspirated in 1 ml syringes and kept on ice until infection.

### Oviduct infections, post challenge treatments and post mortem assessment

Initially all birds were weighed, before the experiments were performed as described by Pors et al. [30]. Briefly, each hen underwent laparotomy under general anaesthesia and received a dose of 0.1 ml of approximately 4×10^4^ CFU injected into the salpinx lumen. To avoid post-surgical pain a semisynthetic opioid, buprenorphine (0.1 mg/kg), was administered to all hens in all experimental groups during surgery, and repeated after 8 and 16 hours.

After completion of the oviduct infections, the forty layers were divided into four treatment groups as shown in Table 1. The individual treatment dose was based on the body weight of the chicken the day of experimental infection. Group one did not receive any further treatment, while all chickens in group 2–4 were each treated once a day in four days, starting 48 hours post challenge. All treatments were given orally. Powder-form of either tetracycline (Doxylin Vet.) (group 2), sertraline (Sertrone) (group 3) or both tetracycline and sertraline (group 4) were dissolved in 1 ml 0.9% NaCl and administered orally to each hen with a dispensable 3 ml plastic transfer pipette.

**Table 1:** Overview of experimental groups. Each group consisted of 10 commercially brown layers, 45 weeks of age.

Group	Treatment	Daily dose/ kg bodyweight (Day 2–5 after challenge)
1	None (infection control)	None
2	Tetracycline	25 mg^1^
3	Sertraline	8 mg^2^
4	Tetracycline +sertraline	25 mg^1^ + 8 mg^2^

^1^ Corresponding to 50 mg Doxylin vet/ kg bodyweight,

^2^Corresponding to 16 mg Sertrone /kg bodyweight

All layers were weighed and euthanized by cervical dislocation seven days post challenge (d.p.i.) and directly submitted to *post mortem* analysis, where lesions were scored according to the scorings system outlined by Pors et al. [30]. Briefly, each organ system (ovary, peritoneum and salpinx) was evaluated based on pre-defined criteria and assigned a score between 0 (no macroscopically pathology) and 4 (extensive macroscopic pathology) for each criteria assessed. The total cumulative score is the sum of scores for all evaluated organ systems in addition to scores on spleen proliferation and lymphatic reaction. Two pathologists scored all lesions of all chickens during the *post mortem* examination, and if any incongruence in scoring occurred, a third pathologist also participated in the scoring process.

From each chicken, sampling from the bone marrow and the cranial and caudal salpinx was done using cotton swabs and plated directly on agar plates (Oxoid, Basingstoke, UK) with 5% bovine blood. Re-isolation was considered positive if abundant growth in pure culture of *E*. *coli* was obtained.

For determination of CFU/ gram salpinx tissue, approximately five centimetres of the middle part of salpinx (magnum) was removed aseptically, weighed off and added to 20 ml 0.9% NaCl solution in a sterile Stomacher filterbag. The contents were homogenised in a stomacher machine for 5 min, and 100 μl of the homogenate was used to make serial 10-fold dilutions in 0.9% NaCl. From each dilution 100 μl was placed on a Müller-Hinton (MH) plate supplemented with 10 mg/ml tetracycline, and distributed equally on the plate using sterile plastic beads. All CFU dilutions were done in duplicate to ensure reproducibility.

Confirmation of re-isolated bacteria as being *E*. *coli* APEC O2 was done by PCR using primers designed in the present study and described below.

### *E*. *coli* APEC_O2 specific primer design

A draft genome sequence of *E*. *coli* APEC_O2 strain was used in this study for *E*. *coli* APEC_O2 strain-specific primer design. pAPEC-O2-ColV and pAPEC-O2-R plasmids were removed from the genome assembly, and the remaining contigs were aligned to 28 genetically-related strains of *E*. *coli* representing sequence types ST135, ST452, ST-15, ST131, ST95, ST140, ST127 and ST73 using progressive MAUVE [31]. Based on the alignment there were 168 regions found to be present specifically in *E*. *coli* APEC_O2. These regions were further checked for similarity to other *E*. *coli* strains by BLAST in non-redundant nucleotide collection of National Centre of Biotechnology Information (NCBI). Hits (n = 46) that were found to be strictly located on a chromosome and/or representing phage sequences were further checked for their similarity to other species in non-redundant nucleotide database, and for the presence of their duplicates in APEC O2. This final step revealed 11 APEC_O2 strain-specific hits, one of which of 217 bp representing phage-related antirepressor was used for primer design using Primer3Plus [32] with the settings for qPCR within the program. Based on this five primer pairs have been selected, and tested against 12 strains of genetically diverse collection of APEC strains described by Ronco et al. [33]. Finally, one primer pair (Forward primer ACCGTTTAGTGCTTCCCAAG; reverse primer ATTGCGACTTCTGTCATGC) with PCR condition including an initial denaturation of 5 min at 95°C followed by 25 cycles of 30 sec at 95°C, 30 sec annealing at 60°C and 30 sec extension at 72°C, and hereafter a finally 10 min of extension at 72°C, yielding a 141 bp PCR product. The primers and PCR condition was proved to be *E*. *coli* APEC_O2 strain-specific in the tested strain collection and was used in this study to confirm re-isolated strains of *E*. *coli* as *E*. *coli* APEC_O2 in the present study.

### Histopathology and immunohistochemistry

For histopathological examination, pieces of the liver and kidney were fixed in 4% neutral buffered formaldehyde solution for 48 h, subsequently trimmed, dehydrated and embedded in paraffin wax prior to preparation of 3 to 5 μm thick sections, which were mounted on adhesive slides (Super Frost/Plus; Menzel-Gläser, Braunschweig, Germany).

Haematoxylin and eosin staining was performed according to a standard protocol [34].

An indirect immunostaining technique based on a specific *E*. *coli* rabbit monoclonal antibody (ab137967; Abcam plc, Cambridge, UK) according to the protocol described by Rossez et al [35] was used for detections of *E*. *coli* in the tissue sections.

### Statistical analysis

For the scoring of pathology, Kruskal–Wallis followed by Dunn’s Multiple Comparison test was used for comparisons of lesion scores. CFU/per gram salpinx and the body weight changes p.i., respectively, one-way ANOVA was used to compare group levels. Subsequently, Tukey's multiple comparison test compared group levels two-by-two for each of the two parameters. All the statistical analysis was done with Graphpad Prism (Graphpad Software, Inc., La Jolla, USA). P<0.05 was considered statistically significant.

## Results

### In vitro synergy assessment

For *E*. *coli* APEC_O2, MICs for tetracycline and sertraline were determined to 64 mg/L and 32 mg/L, respectively (Table 2). Synergy between tetracycline and sertraline as defined by FIC ≤ 0.50, could be obtained for *E*. *coli* APEC_O2 at an exposure of 8 mg/L tetracycline combined with 16 mg/L sertraline.

**Table 2:** Minimal inhibitory concentration (MIC) of sertraline and tetracycline alone or combined. For each combination exposure on sertraline and tetracycline, the fractional inhibitory concentration index (FICI) is stated.

MIC		FICI
Tetracycline (μg/ml)	Sertraline (μg/ml)	
0	32	-
64	0	-
32	0.25	0.96
16	8	0.50
4	16	0.56

### Pathological outcome of infection

**Fig 1:**
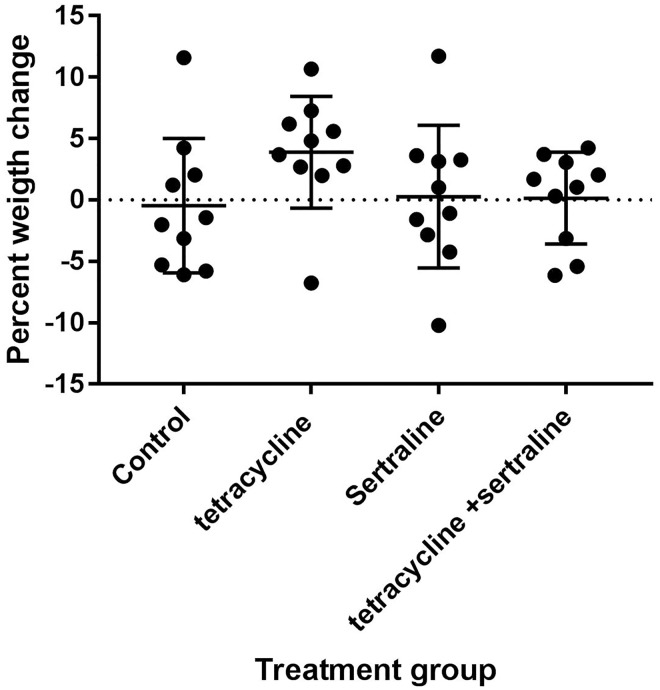
Weight changes during infection. Percent weight change seven days after infection compared to before infection per chicken in the different treatment groups.

The surgical procedure of infection was conducted in an uncomplicated manner for all chickens. There were no mortality in any of the groups at day seven following challenge, and besides a slight depression observed in all groups 12–24 hours after challenge, all chickens displayed normal avian behaviour, and normal drinking and feeding activities, and the average weight change per chicken did not differ significantly between groups (Fig 1).

**Fig 2:**
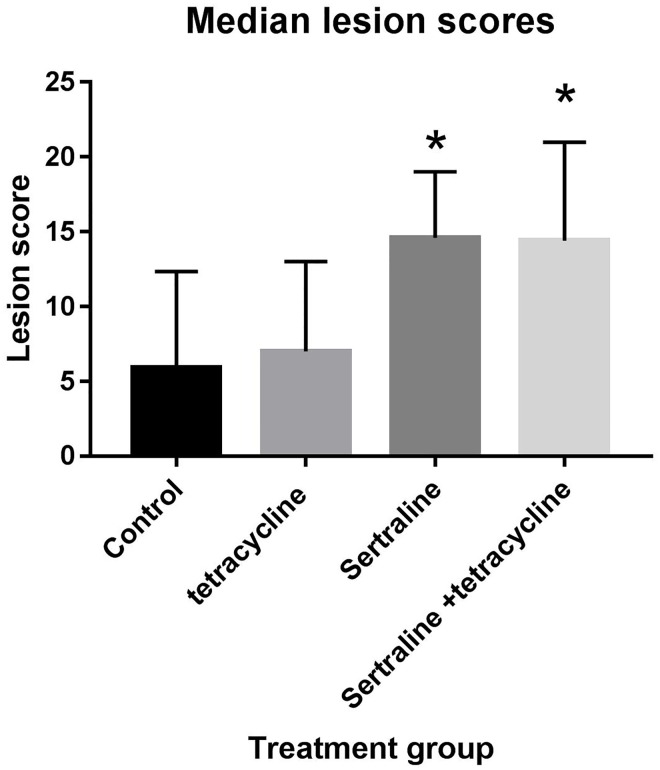
Lesion scores. Median lesion score per chicken in the different treatment groups. Vertical bars indicate standard deviation, and asterisk indicated statistical difference in lesion score compared to the non-treated control group.

At *post mortem* examination seven days post challenge, large variations in the pathological outcome was observed (Fig 2). Overall, irrespective of post infection treatment gross macroscopic observation consisted of varying degrees of peritonitis, salpingitis and oophoritis. The least severe pathology as defined by lowest lesions score were observed among chickens in the untreated control group, although the median score did not differ significantly from the median lesion score of chickens in the tetracycline-treated group. For the untreated group of chickens, 6/10 did not demonstrate any pathology at all (lesion score 0), while 5/10 chickens in the tetracycline-treated group all had lesion score of 5 or lower.

When compared to the untreated and tetracycline-treated groups, the pathology observed in the remaining groups (sertraline and sertraline/tetracycline) was considerably more severe and productive/suppurative, and only a single chicken had a lesion score below 10 in the sertraline-treated group, and for the sertraline/tetracycline treatment group, 3/10 chickens had a lesion score greater than 20. The median lesion score in both the sertraline and sertraline/ tetracycline treatment group were significantly higher than the control group (*P*< 0.011 and *P*<0.014, respectively).

### Histopathology and immunohistochemistry

**Fig 3:**
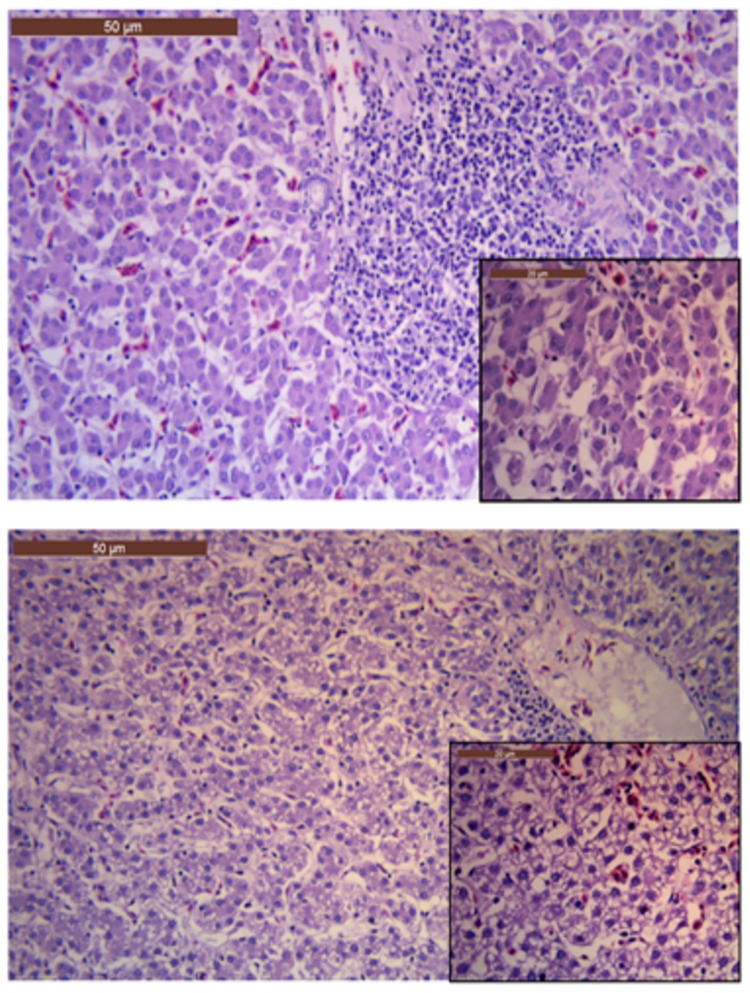
Liver histology. a) Liver section from a chicken in the control group (not receiving any treatment after challenge with *E*. *coli* APEC_O2). Notice the normal and regular hepatocytes; b) Liver section from a chicken in the sertraline-treated group. Notice the major vacuolization of the hepatocytes. Each of the large images is at 20X magnification, insert in the corners are from the same image, but at 40X magnification.

Similar to the macroscopic variations observed, the histopathological observations also ranged from no histopathological findings to severe vacuolisation of the hepatic cells in the different groups (Fig 3). Severe vacuolisation of the hepatic cells were solely found in liver sections from chickens in the sertraline- or sertraline/tetracycline groups. No histopathological findings were observed in the kidney sections, irrespectively of treatment groups. Positive staining for *E*. *coli* was not observed in any of the tissue samples.

### CFU of the salpinx

**Fig 4:**
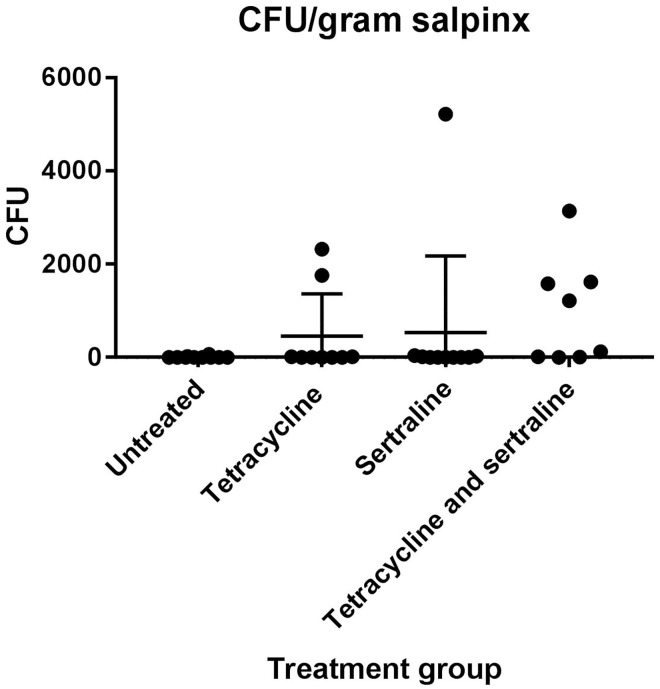
Colony-forming units (CFU). CFU per gram salpinx in the different treatment groups. Vertical bars indicate the standard deviation.

For all chickens, CFU of *E*. *coli* APEC_O2 in approximately 20 gram of the salpinx was determined. There was no difference between the average CFU/g salpinx per chicken between groups (p = 0.171) (Fig 4).

The weight of each chicken was measured before and seven days after challenge. There were no statistical differences in the average weight changes per chicken between groups (Fig 1).

## Discussion

In humans, urinary tract infections (UTI) due to *E*. *coli* are one of the most common reasons for antibiotic treatment in clinical practise [36]. Emergence of multi-resistant *E*. *coli* will consequently have major impact of treatment success of UTI in human medicine. Similar, in poultry *E*. *coli* is the most frequent cause of ascending infections, and accumulating evidence suggest that the clones of *E*. *coli* causing ascending infections in poultry are highly similar to clones of *E*. *coli* causing UTI in human [23,37]. In the present study we have assessed the potential of sertraline as antimicrobial or as an antimicrobial helper compound in the treatment of experimental ascending *E*. *coli* infections in poultry.

In the study, the strain *E*. *coli* APEC_O2 was used as challenge strain. The strain was chosen because it is a tetracycline resistant avian pathogenic *E*. *coli* and because it has been shown to possess a plasmid enhancing survival in human urine and increasing colonization of the murine kidney [27]. The strain has, however, never been used to experimentally infect the salpinx of poultry, therefore the virulence potential in this aspect was unknown prior to the present study. In the infection control group (Group 1) only 4/10 chickens had recognisable macroscopic pathology, while the remaining six chickens in the group showed no signs of gross pathology. Out of these six macroscopically unaffected chickens, *E*. *coli* APEC_O2 could not be re-isolated from the tissue either (Fig 3). The host-related factors resulting in varying susceptibility is well-documented, although it could be argued if the infection dose used was too low [30]. Ideally, 10/10 chickens in the untreated group would had shown gross pathology consistent with an ascending *E*. *coli* infection. However, previous studies have documented that host-related factors, the infection dose and strain-related characteristics are important parameters for infection outcome [38]. In a recent study, applying the same method, but using another strain of *E*. *coli* as challenge, a 100% mortality two days post challenge was observed when applying a dose of 5x10^5^ CFU [39]. To optimize the chances for full survival of all chickens in all groups, while still ensuring an infection to be established in at least part of the chickens, the dose was set two log units lower than in the previously mentioned study.

As *E*. *coli* APEC_O2 is tetracycline resistant, it was not surprising that the pathology in the tetracycline-treated group was highly similar to the observations done in the un-treated control group. The CFU per gram salpinx were also similar with the counts in the control group, although two chickens had CFU counts significantly higher than for any of the chickens in the control group (Fig 4), indicating clearly that *E*. *coli* APEC_O2 is not inhibited by tetracycline, unless administered in concentrations eight times higher than the cut-off value of tetracycline sensitivity of *E*. *coli* as defined by EUCAST[40] (Table 2).

In contrast to all other groups, all but one chicken in the tetracycline-treated group, actually had gained weight in the week following infection (Fig 1). If this is due to ease of infection, and consequently greater appetite, or a better feed conversion rate when tetracycline is administered as suggested by several studies [41,42] is unknown as all groups were feed *ad libitum* with no records on actual feed intake, but anorexia/decrease in appetite was not observed in any of the groups during the trial.

At seven days post challenge, the bone-marrow of all chickens from all groups was culture negative, indicative of the lack of a septic/bacteraemic condition. In agreement *E*. *coli* was not detected in any of the histopathological slides. The severity of the macroscopic pathology observed for chickens in sertraline and sertraline/tetracycline treated groups was highly similar, and more pronounced than in the un- or tetracycline- treated groups (Fig 1). The infections were considerably more productive and more extensive cases of peritonitis involving the entire peritoneal cavity were observed. The histopathology revealed the chickens in these groups also had marked vacuolisation of the hepatic cells (Fig 3), a finding previously associated with infectious diseases in avian species [43]. In contrast, the level of CFU in salpinx was statistically indifferent from counts in the control group although there was a tendency to higher counts among chickens in the sertraline/tetracycline treated group (Fig 4). Based on the *in vitro* synergy assessment assay, it was evident that a relative high concentration of sertraline is needed to reduce the tetracycline resistance of *E*. *coli* APEC_O2 to 8 mg/L tetracycline, the epidemiological cut-off for resistance [40]. As sertraline is known to have a low toxicity [44], and due to the *in vitro* observations (Table 2), a daily dose of 8 mg sertraline/kg body mass chicken was considered as a relevant experimental dose, which is approximately 2.5 times the concentration used to treat severe clinical depression in human medicine [45].

The more severe outcome of infection in the sertraline/tetracycline group was unexpected, as it was hypothesized to significantly improve/combat the infection. An explanation for the unexpected discrepancy of the pathology and CFU counts when compared to control group was sought for in the literature. As to the authors knowledge this is the first time sertraline treatment in chickens in an infectious model has been described. In behavioural (non-infectious) avian studies, exposure of SSRI compounds in concentrations from 5.0 mg/L [46] to 25 mg/L [47] have been published with no reports on observed pathology/toxically when SSRIs are used in poultry. Neither did the histopathological examination in the present study show any signs of increased toxicities. There were, however, signs of exacerbated infection (liver vacuolisation), indicating the bacterial infection rather than the compound of sertraline per see worsened the outcome. These observations may lead to the tentative conclusion that the sertraline compromise the innate immune system of the chickens. In humans, SSRIs has been documented to suppress lymphocyte proliferation, cytokine secretion and viability *in vitro*, although the mechanism behind SSRI-induced immunological effects remains to be elucidated [48]. The finding that SSRIs suppress unwanted immune reactions has been demonstrated in animal models of autoimmune disorders, such as rheumatoid arthritis [49] and multiple sclerosis [50]. Furthermore, a beneficial effect of SSRIs in the treatment of lymphoma has been proposed, presumably attributed to both a direct suppressive effect on the malignant cells and a stimulatory effect on anti-cancer immunity [51,52]. Finally, recent evidence showed an effect of fluoxetine, another SSRI compound, on neutrophil adhesion and recruitment to inflammatory sites, demonstrating that not only cellular but also innate immunity is impacted by SSRIs [53].

Only few studies have investigated the interaction of SSRI and bacterial infection, experimentally induced [54] or clinical observations [55]. For the latter, a recent study documented that there is an increased risk of acquiring a *Clostridium difficile* infection when taking daily doses of SSRI [56,57], despite that it is generally assumed that the required doses for immunosuppression has been suggested to be higher than those used to treat depression [48]. In contrast, relatively low concentrations (0.1–1 μM) of a SSRI compound, fluoxetine, have been found to stimulate T cell proliferation [58].

The avian immune system resembles that of mammals since both evolved from a common reptilian ancestor and have inherited many commonalities [59]. They have also developed a number of different strategies that are unique to birds. The lymphoid organs plays a major role in avian immunity, with the bursa of Fabricius (site of B-cell origin) and the thymus (site of T-cell origin) are considered primary lymphoid organs [60]. Since T-cells are suggested to be particular sensitive to high-doses sertraline [61], it may be speculated that avian species are particular susceptible to SSRI-induced immunosuppression.

In conclusion, although sertraline has a synergic effect with tetracycline *in vitro*, combinational treatment with sertraline and tetracycline significantly worsen the pathological outcome of infection in an experimental bacterial infection model. These findings may be explained by an immunosuppressive effect of sertraline when applied in doses four times that of clinical doses recommended for treatment of central nervous system disorders in man.

A possible beneficial effect of lower doses of sertraline in combination with tetracycline treatment of tetracycline resistant *E*. *coli* infections requires future studies to answer.

## Supporting information

S1 Table All raw data. (XLSX)
